# Discontinuous phase diagram of amorphous carbons

**DOI:** 10.1093/nsr/nwae051

**Published:** 2024-02-06

**Authors:** YinBo Zhu, ZhouYu Fang, ZhongTing Zhang, HengAn Wu

**Affiliations:** CAS Key Laboratory of Mechanical Behavior and Design of Materials, Department of Modern Mechanics, University of Science and Technology of China, Hefei 230027, China; CAS Key Laboratory of Mechanical Behavior and Design of Materials, Department of Modern Mechanics, University of Science and Technology of China, Hefei 230027, China; CAS Key Laboratory of Mechanical Behavior and Design of Materials, Department of Modern Mechanics, University of Science and Technology of China, Hefei 230027, China; CAS Key Laboratory of Mechanical Behavior and Design of Materials, Department of Modern Mechanics, University of Science and Technology of China, Hefei 230027, China; State Key Laboratory of Nonlinear Mechanics, Institute of Mechanics, Chinese Academy of Sciences, Beijing 100190, China

**Keywords:** amorphous carbons, disordered carbons, phase diagram, microstructural topology, phase transition

## Abstract

The short-range order and medium-range order of amorphous carbons demonstrated in experiments allow us to rethink whether there exist intrinsic properties hidden by atomic disordering. Here we presented six representative phases of amorphous carbons (0.1–3.4 g/cm^3^), namely, disordered graphene network (DGN), high-density amorphous carbon (HDAC), amorphous diaphite (a-DG), amorphous diamond (a-D), paracrystalline diamond (p-D), and nano-polycrystalline diamond (NPD), respectively, classified by their topological features and microstructural characterizations that are comparable with experiments. To achieve a comprehensive physical landscape for amorphous carbons, a phase diagram was plotted in the sp^3^/sp^2^ versus density plane, in which the counterintuitive discontinuity originates from the inherent difference in topological microstructures, further guiding us to discover a variety of phase transitions among different amorphous carbons. Intriguingly, the power law, log(sp^3^/sp^2^) ∝ *ρ*^n^, hints at intrinsic topology and hidden order in amorphous carbons, providing an insightful perspective to reacquaint atomic disorder in non-crystalline carbons.

## Teaser

A phase diagram for amorphous carbons was plotted in the sp^3^/sp^2^ versus density plane, where counterintuitive discontinuity originates from inherent differences in topological microstructures, further guiding us to discover various phase transitions.

## INTRODUCTION

Amorphous solids only exhibit localized order at small length scales [1], possessing excellent multifaceted physical and mechanical properties compared to their corresponding crystalline counterparts. Due to their structural intricacies, amorphous carbons are one kind of the most elusive elementary substances. Different hybridizations of C**−**C bond (sp, sp^2^, and sp^3^) endow carbon allotropes with various forms and surprising properties. In the 1940 s, glassy carbon had been discovered by Rosalind E. Franklin in carbonized coals [2]. Seminal insights given by Franklin have spurred enormous interest and substantial advances in the field of glassy carbons and other amorphous carbons [3–7]. The absence of long-range order in amorphous carbons renders it notoriously difficult to identify their microstructures [8,9]. Current characterization techniques of amorphous carbons are very limited, such as electron energy loss spectroscopy (EELS), X-ray diffraction (XRD), and structural factor (S(Q)), which are insufficient to give clear microstructural landscapes. High-resolution transmission electron microscopy (HRTEM) images can only recognize a few ordered atomic patterns with ∼1 nm characteristic size in fog-like vague microstructures. Although locally ordered features in amorphous carbons have been experimentally demonstrated [10], the atomic arrangement remains an open issue.

Recently, synthesis of new amorphous carbons extended the knowledge of atomic disorder in materials [11]. Özyilmaz and coworkers reported monolayer amorphous carbons with ring distributions of the competing crystallite model [12] topologically distinct from conventional disordered graphene. Most amorphous carbons synthesized in experiments are three-dimensional (3D) carbon allotropes without the long-range order, including graphene-based disordered carbons, pyrolytic carbons, glassy carbons, and diamond-like carbons. Tour and coworkers demonstrated, in gram-scale flash graphene [13], turbostratic arrangement between stacked graphene layers. Tang *et al.* and Shang *et al.* synthesized different millimeter-scale forms of atomically disordered diamonds [14,15], in which the medium-range order was evidenced by ∼1 nm diamond-like nuclei in HRTEM images. However, a comprehensive physical image of different 3D amorphous carbons is still lacking. The disorder in amorphous carbons comes from the significant differences in atom-scale microstructural topologies comprised to crystalline allotropes. It is hard to classify different amorphous carbons due to their mysterious yet unidentified topology. Given that short-range order and medium-range order are found in amorphous carbons, a question arises whether amorphous carbons exhibit intrinsic properties hidden by disordering, further allowing us to reacquaint the topology and hidden order in atomically disordered carbons.

In what follows, we performed large-scale molecular dynamics (MD) simulations to present comprehensive physical insights for 3D amorphous carbons with densities ranging from 0.1 to 3.4 g/cm^3^. The environment-dependent interaction potential (EDIP) developed by Marks was used [16,17], which has proven to be a powerful forcefield to explore amorphous carbons [18–20]. The topological microstructures of six representative phases are demonstrated, which are in line with experimental characterizations of amorphous carbons. More importantly, a phase diagram of amorphous carbons is plotted in the sp^3^/sp^2^ versus density plane, in which the counterintuitive discontinuity originates from the inherent difference of microstructural topology. This phase diagram then guides us to discover and understand transformations among different amorphous carbons.

## RESULTS AND DISCUSSION

**Figure 1.**
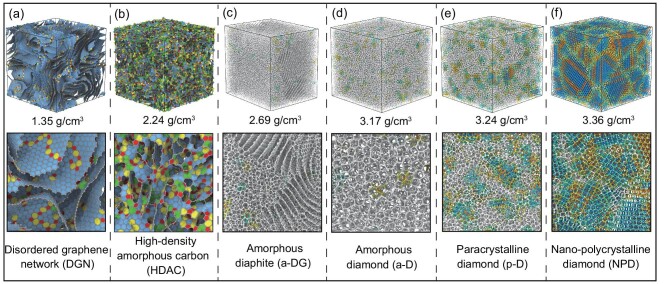
Six representative phases of simulated amorphous carbons. (a) DGN. (b) HDAC. (c) a-DG. (d–f) Three different ta-Cs: (d) a-D, (e) p-D and (f) NPD, respectively. Simulation scale of each model is ∼108 000 atoms. The upper row shows 3D views of each amorphous carbon, and the bottom row shows local slices of corresponding phase. More details of microstructural topology and color index are shown in Figs S1–S4.

Figure 1 plots six representative amorphous carbons obtained from our large-scale MD simulations. In Fig. 1a, disordered graphene network (DGN) features a disordered 3D network of graphene nanosheets [21], which well exhibits continuous 3D connectivity and high graphitization. DGNs with varied densities (*ρ* < 2.4 g/cm^3^) can thus be used to model disordered carbons [21–23], including porous carbon, pyrolytic carbon, hard carbon, and amorphous graphite. Martin *et al.* demonstrated that the topology of DGNs is mainly in the negative-curvature microstructures [21], in combination with saddle-shaped graphene nanosheets with out-of-plane topological defects [24]. The fraction of sp^2^ bonds in all DGNs is larger than 97%, which is much closer to experimental measurements utilized by EELS spectra [25]. From the perspective of structural ordering, short-range disorder in DGNs is mainly the in-plane defects (5–7 defects and defect clusters) while long-range disorder originates from non-oriented nanosheets (Fig. S1). The high-density amorphous carbon (HDAC) in Fig. 1b exhibits a highly disordered microstructure, which seems to be a close-cell material composed of nanoscale but turbulent fragments (Fig. S2). Each nanoscale fragment in HDACs can be regarded as a cell wall with the characteristic size of several carbon rings, while local microstructures are more distorted due to hybrid sp^2^-sp^3^ nanoforms [26]. In simulated HDACs, the density ranges from 1.8 to 2.5 g/cm^3^, and the fraction of sp^3^ bonds is 17%–34%. The structural ordering only exhibits the short-range order, in which hexagonal carbon rings are the main part of fragments (Fig. S2). Parallel fragments in HDACs can be regarded as nanometer-sized graphite-like nuclei embedded into disordered carbons, which is reminiscent of the widely-used simplified model of glassy carbons [10,26]. Diaphite, a hybrid structure of diamond and graphite has been discovered in meteorites [27]. In Fig. 1c, amorphous diaphite (a-DG) exhibits hybrid features of amorphous graphite and atomically disordered diamond, in which the local graphite microstructure endows a-DG with the medium-range order. The microstructural features of a-DGs plotted in Fig. S3 are similar to the C/C composites reported by Li and coworkers [28]. Here a-DG is difficult to obtain directly from annealing-MD simulations due to the hybrid microstructures, while it can be obtained in phase transformations of DGNs. The fraction of sp^3^ bonds (18%–69%) in a-DGs increases with the increase of density (2.55–3.16 g/cm^3^).

Atomically disordered diamonds (*ρ* > 3.0 g/cm^3^) have been synthetized recently under extreme experimental conditions [14,15,29–31]. In simulations, we found three different tetrahedral amorphous carbons (ta-Cs) namely: amorphous diamond (a-D, Fig. 1d), paracrystalline diamond (p-D, Fig. 1e), and nano-polycrystalline diamond (NPD, Fig. 1f), respectively. Here a-D and p-D are similar to recently reported atomically disordered diamonds obtained from the well-equilibrated C_60_ precursor [14,15], and thus we use the same nomenclatures as referred in the work of Gou and coworkers [14]. It should be noted that our simulation method is more general and easier to operate, which can avoid the requirement of different simulation methods to obtain a-D and p-D. In Fig. 1d–f and Fig. S4, apparent differences are the fraction and characteristic size of diamond-like nanonuclei (paracrystallites) [14]. For a-D, the fraction of sp^3^-bonded atoms within paracrystallites is lower than 10% (Fig. S4d), resulting in most atoms being in the disordered state without medium-range order [6,30]. For p-D, many paracrystallites emerged, endowing p-D with the medium-range order [14]. While those paracrystallites distribute randomly, most sp^3^ bonds in the first- and second-neighbor of paracrystallites are in the amorphous state. The cross-section profiles of simulated p-D are similar to HRTEM images of atomically disordered diamonds [14]. In experiments [14,15], diamond-like clusters with the medium-range order were found without regular distributions, indicating random but not yet understood nucleation events during the formation of p-D. Figure S5 illustrates nucleation snapshots in liquid carbon. The temperature-dependent paracrystalline nucleation in atomically disordered diamonds was revealed in our recent metadynamic simulations [31]. In NPD (Fig. S4c), large-size diamond nanograins are found, separated by disordered sp^3^-bonded atoms. Nanograins in NPDs are inhomogeneous, and both cubic and hexagonal diamond phases coexist randomly [32]. The density range of p-D (3.2–3.29 g/cm^3^) is overlapped with a-D (3.1–3.24 g/cm^3^) and NPD (3.23–3.39 g/cm^3^) (Fig. S6). In simulations, we found that high temperature and high pressure can increase the fraction of sp^3^ bonds. Under high temperature, a certain range of high pressure can increase paracrystallites. During the quenching process, slightly higher pressure can help to increase the volume fraction of paracrystallites. But, coordinated high-temperature and high-pressure conditions as well as other factors still need to be clarified [14] since it is hard to control nucleation of disordered paracrystallites. Our simulations independent of the C_60_ precursor also indicate the inherent existence of a-D and p-D phases in amorphous carbons.

**Figure 2.**
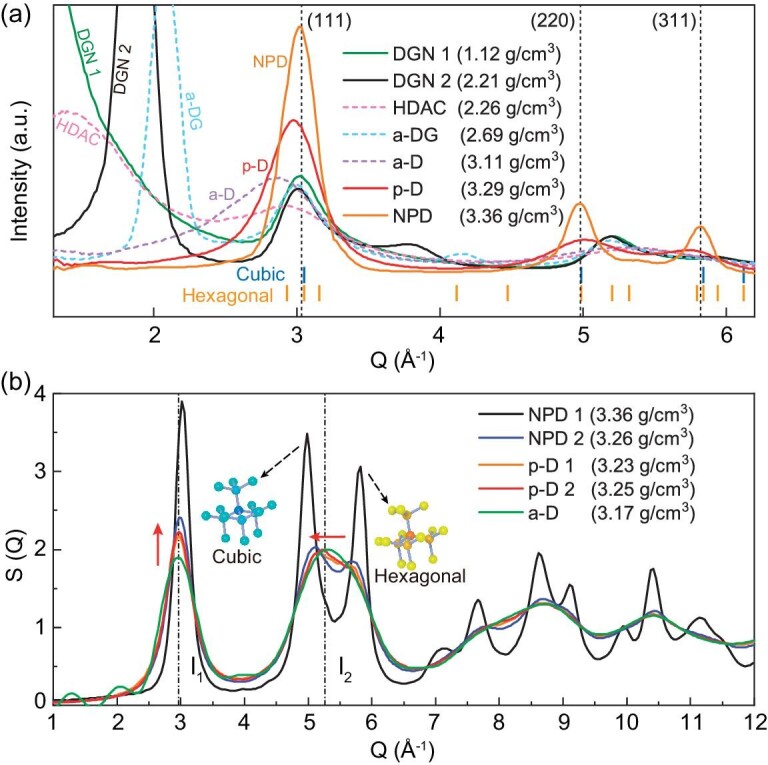
Identification of different amorphous carbons. (a) Computed XRD spectral curves of different amorphous carbons. The short vertical lines denote diffraction peaks of cubic and hexagonal diamonds. (b) Structural factors. The height difference between I_1_ and I_2_ can be used to distinguish a-D and p-D [6,14]. The differentiated I_2_ peak indicates the arising of diamond-like nanograins. The computed XRD and S(Q) are comparable with recent experimental measurements of amorphous carbons [6,14,15,25,26,28,29,33].

Subsequently, we used the computed XRD and S(Q) to characterize different amorphous carbons. The broad peaks in XRD curves indicate missing long-range order. In Fig. 2a, p-D and NPD exhibit obvious characteristic peaks at (111), (220) and (311) which are the diffraction peaks of diamond. While a-D only exhibits a broadened peak at ∼2.9 Å^−1^, hinting at less ordered sp^3^-bonded atoms. In addition to a broadened peak at ∼3.0 Å^−1^, a-DG exhibits an obvious peak at ∼2.1 Å^−1^, indicating graphene multilayers [28]. Computed XRD patterns of DGNs have been well discussed in a previous study [21]. HDAC exhibits much broadened peaks located at ∼1.6 and ∼3.0 Å^−1^, bearing a considerable resemblance to experimental XRD patterns of glassy carbons [33]. The S(Q) in Fig. 2b can identify the subtle but definite differences among a-D, p-D, and NPD [6,14]. With the increase in density, the height of I_1_ peak increases while I_2_ peak appears differentiation. For a-D, I_1_ peak is lower than I_2_ peak [6], which is in contrast with p-D [14]. The differentiated I_2_ peak of p-D is inconspicuous, since finite-sized paracrystallites only occupy a small fraction of the whole structure. The S(Q) results of a-D and p-D are consistent with experimental data [6,14], indicating that our simulated a-D and p-D based on EDIP are reasonable and reliable. We also computed radial distribution functions, coordination number, and distributions of bond angle and bond length (Fig. S7) of different amorphous carbons. Especially, in Fig. S7b, the coordination number of each amorphous carbon exhibits a specific approximation, implying that fractions of sp^3^ and sp^2^ bonds should be emphasized when we concern the topology of amorphous carbons [32].

**Figure 3.**
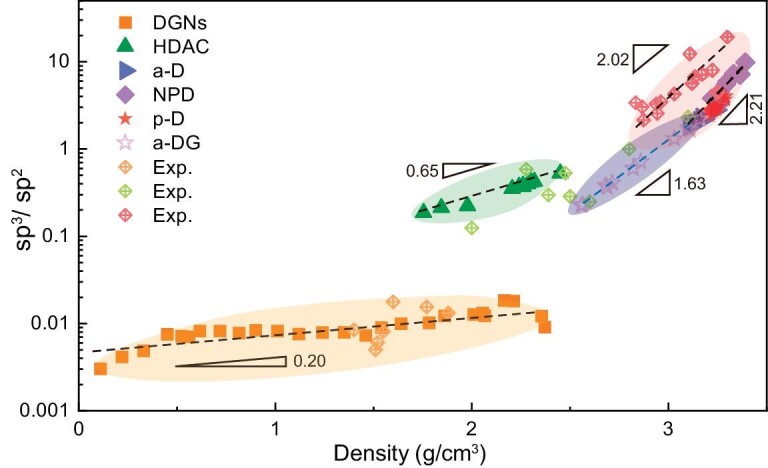
Phase diagram of amorphous carbons. The discontinuous feature of this phase diagram originates from the inherent difference of microstructural topology highlighted in Fig. 1 and Figs S1–S4. The dashed lines indicate inherent scaling laws between the sp^3^/sp^2^ and density. The experimental data are listed in Table S3.

To present a comprehensive physical image for amorphous carbons, we summarized simulation data and previous experimental data in Fig. 3. Here the sp^3^/sp^2^ value is used as an important physical quantity to distinguish amorphous carbons. The sp bond is not considered here due to its very small fraction (<3.5%), which can mainly be found in DGNs and HDACs (for example, in Fig. 1a and b the fractions of sp bonds are only 1.1% and 1.65%, respectively). Counterintuitively, this phase diagram is discontinuous, in which two main gaps indicate there are intrinsic differences between different amorphous carbons. In experiments, the absence of atomic-scale microstructural characterizations brings the question of how to classify different amorphous carbons and whether they have intrinsic properties, though similar features of amorphous carbons within a certain density range have been reported previously [3–7,13–15,21–33]. The dashed lines in Fig. 3 give the fitted power law between sp^3^/sp^2^ (logarithm) and density for different amorphous carbons, hinting at an intrinsic topology hidden in their disordered microstructures. This allows us to rethink the atomic disorder in amorphous carbons. More importantly, this discontinuous phase diagram presents an insightful perspective that connotes many important intrinsic properties and relationships hidden in non-crystalline carbons. In what follows, we focus on the microstructural topology and phase transformation of different amorphous carbons.

Determining the microstructural topology is of significance to non-crystalline carbons [21,32]. We here combine Fig. 1 with Fig. 3 to depict the topology of different amorphous carbons. DGNs feature a disordered network with high graphitization (sp^3^/sp^2^ < 0.01), in which sp^3^ bonds are contributed by out-of-plane topological defects to ensure 3D connectivity [24]. The density range of DGNs is wide, including low-density porous carbons (ρ < 0.6 g/cm^3^), medium-density disordered carbons (0.6–1.8 g/cm^3^), and amorphous-graphite carbons (1.8–2.4 g/cm^3^). The fitted power law for DGNs is log(sp^3^/sp^2^) ∝ *ρ*^0.2^, indicating that all DGNs follow a similar microstructural topology as plotted in Fig. 1a and Fig. S1. The medium-range order in DGNs is featured mainly by graphene nanosheets with few in-plane defects, while the missing long-range order originates from the disordered network connected by out-of-plane topological defects. In HDACs, local parallel fragments form nanometre-sized amorphous graphite (short-range order), embedded into the disordered microstructure through distorted out-of-plane topological defects. The fitted power law for HDACs is log(sp^3^/sp^2^) ∝ *ρ*^0.65^ and sp^3^/sp^2^ <0.6, indicating that amorphous sp^2^-bonded atoms occupy the main fraction [26]. With increasing density, HDACs exhibit more sp^3^ bonds but the topology remains the highly disordered microstructure with only the short-range order as plotted in Fig. 1b and Fig. S2. In a-DGs, distinguishable graphene multilayers provide the medium-range order [28], though the fraction of sp^3^ bonds exhibits a large range (18%–69%). The fitted power law for a-DGs is log(sp^3^/sp^2^) ∝ *ρ*^1.63^, indicating that all a-DGs exhibit the same microstructural topology as plotted in Fig. 1c and Fig. S3. The medium-range order in a-DGs is featured mainly by the multilayer graphene and diamond-like nuclei. For ta-Cs, the sp^3^/sp^2^ value of simulated structures is smaller than that of experimental data, in which the fraction of sp^3^ bonds in simulations is 5%–15% lower than that estimated from EELS in experiments [14,15]. This tolerable difference should be attributed to the limitation of EDIP in the simulations of highly sp^3^-bonded carbons [17,31], more effort in finding coordinated high-temperature and high-pressure conditions should be devoted to simulations to improve the fraction of sp^3^ bonds [34]. However, the fitted power law of experimental data (log(sp^3^/sp^2^) ∝ *ρ*^2.02^) is much close to that of simulation results (log(sp^3^/sp^2^) ∝ *ρ*^2.21^), indicating that the simulated microstructural topology (Fig. 1d–f and Figs S4 and S5) is similar to their experimental counterparts. In a-D, p-D, and NPD, the short-/medium-range order depends strongly on the paracrystalline nucleation [31]. The overlapped density ranges in Fig. S6 imply that p-D is easily enshrouded by a-D and NPD, while p-D can be identified through the S(Q). Our recent study presented a clear landscape of the formations of a-D, p-D, and NPD affected by different temperatures [31], which can answer why p-D arises distinctly from other ta-Cs. Thus, the proposed power law, log(sp^3^/sp^2^) ∝ *ρ*^n^, can be used to identify different amorphous carbons. The index of ‘n’ in this power law also indicates that, in some intervals of density, the microstructural stability of amorphous carbons can be mediated through the change of sp^3^/sp^2^ associated with suitable pressure-temperature conditions during phase transformation. It is expected that, behind the microstructural disorder, more interesting properties (e.g. mechanics and thermodynamics) of amorphous carbons will be discovered in the future.

Although EDIP is an empirical potential [16–20], structural analyses in Fig. 2 and Fig. S7 are comparable with experimental measurements. Previous studies also proved the EDIP force field with excellent abilities in predicting different amorphous carbons [17–24,31]. Thus, Fig. 3 can provide a reliable framework and forward-looking landscape for amorphous carbons within a large density range. It is also anticipated that more powerful force fields (e.g. machine-learning potential) will be developed to depict the phase diagram more precisely. Various topological defects are non-negligible when we analyze microstructures of amorphous carbons. In monolayer amorphous carbons [12,35–37], it was found that the coexistence of a large fraction of crystallite domains and random defective regions seems to be necessary for stability. In blocky amorphous carbons, topological defects served as matrix (or junctions) to connect randomly distributed crystallites and cooperatively form reasonably disordered microstructures [21–24,28–31], resulting in the missing of long-range order. Amorphous carbons obtained from large-scale MD simulations can contain more topological features than the small-size atomic configurations predicted from first-principles calculations. Our simulated disordered microstructures are approximately isotropic due to the large box size (>100 000 atoms). The size of our simulations can be extended to the scale of 1 million carbon atoms, and the main topological microstructures are mainly the six types. We also noted that steric inhomogeneity can result in different phases with the same density, which is not considered in our phase diagram. For example, Fortunelli and coworkers obtained diverse sp^2^-rich amorphous carbons from stochastic simulations [38], in which low-density phases (porous carbons) exhibit different configurations due to the steric inhomogeneous networks. More importantly, this discontinuous phase diagram provides a framework for establishing a unified understanding of the microstructural topologies of amorphous carbons, spurring us to ponder over phase transformations and possible pathways in future experimental syntheses [11]. In the next paragraph, inspired by the discontinuous phase diagram, we presented several typical phase transitions among different amorphous carbons.

**Figure 4.**
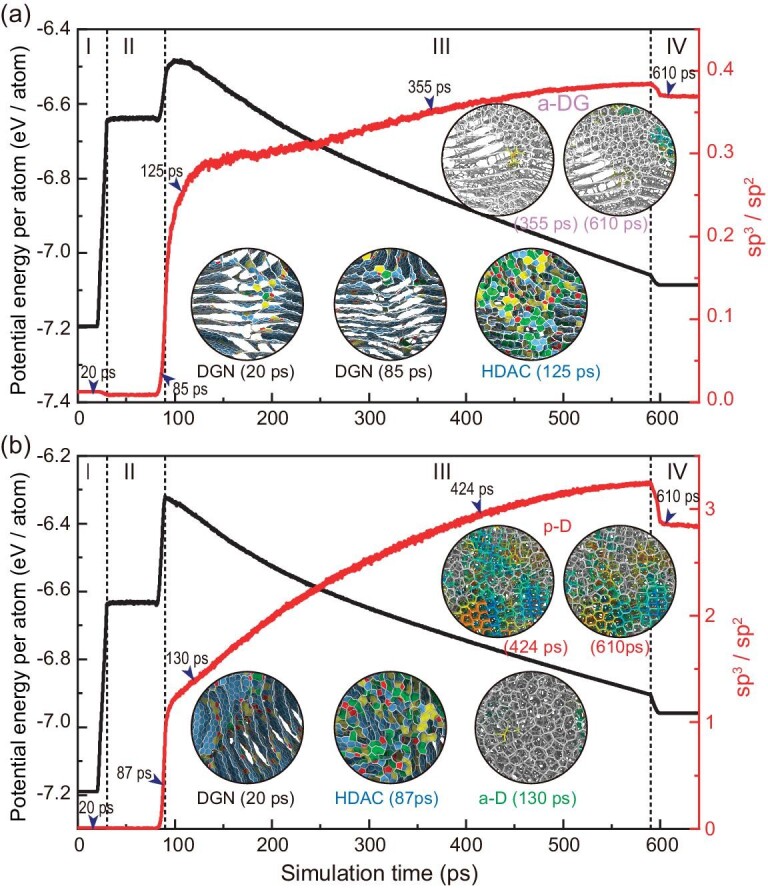
Phase transitions among different amorphous carbons. (a) Transformation from DGN to a-DG. (b) Transformation from DGN to p-D. The insets demonstrate corresponding microstructural evolutions. Simulation conditions are listed in Table S2.

In Fig. 4a, starting from a DGN precursor, a-DG is finally obtained through controlling temperature and pressure (Table S2). During the phase transition, HDAC as a transient phase arises at the beginning of cooling process, indicating that high temperature and high pressure can destroy the stable in-plane sp^2^ bonds. Then, in stage III (4000 to 300 K), graphitization of in-plane sp^2^ bonds and nucleating growth of amorphous sp^3^-bonded atoms finally trigger the appearance of a-DG [28]. This DGN**−**to**−**HDAC**−**to **−** a-DG transformation further inspired us to explore how to obtain a-D and p-D through phase transformations independent of a C_60_ precursor. Recent experiments suggested that the synthesis of diamond-like amorphous carbons needs high temperature and high pressure [14,15]. In Fig. 4b, the applied pressure in stage II was thus set to be 40 GPa (Table S2). As expected, stable p-D was finally obtained. The local stacked graphene layers in DGNs were destroyed by ultrahigh pressure, resulting in the arising of unstable HDAC at the end of stage II. Then, unstable a-D phase was found in the following cooling process. After long-time annealing, heterogeneous nucleation of amorphous sp^3^-bonded atoms resulted in p-D formation. Under the framework of Arrhenius theory proposed by Marks and coworkers [19], the simulated high temperature can be associated with the experimental temperature. Thus, the transformation from DGN to p-D in Fig. 4b should present another promising method to synthesize diamond-like amorphous carbons (e.g. p-D). Herein, the high temperature of 4000 K used in our simulations is to promote the occurrence of phase transitions and reduce the simulation time. In our recent study [31], the effect of temperature on paracrystalline nucleation in ta-Cs was revealed by using metadynamics and two collective variables. We found that p-D was preferred in a narrow range of temperatures, while unsuitable temperatures would result in the formation of a-D or NPD. Apparently, temperature plays a vital role in the formation and transformation of different amorphous carbons. It is expected that a clear landscape of temperature effect can help us gain more in-depth insights into the phase transitions of various amorphous carbons.

Last but not least, it is interesting to note that the overlapped density range of DGNs and HDACs in Fig. 3 prefigures reversible phase transformations between high-density DGNs (amorphous graphite, Fig. S1c) and HDACs. In the density range of 1.8–2.4 g/cm^3^, the phase diagram indicates that amorphous carbons exist in two distinguishable phases (amorphous graphite and HDAC) concurrently, which can explain the recently reported transitions between disordered carbon and amorphous graphite in *ab initio* simulations [39]. To verify this reversible transition, HDAC was selected as the precursor. In Fig. S8, potential energy in stage II (200 ps equilibrium run under 4000 K) exhibits a large drop. The spontaneous transformation from HDAC to amorphous graphite could be attributed to the low fraction of sp^3^ bonds that are sensitive to high temperature, resulting in the breakage of sp^3^ bonds and the following graphitization of sp^2^-bonded atoms. It can also be deduced that DGN exhibits a relative lower energy state compared to HDAC [39]. Then, the applied high pressure (40 GPa) reverses the phase transition, manifesting the reappearance of HDAC. It can be deduced that high pressure contributes to damage of in-plane sp^2^ bonds in amorphous graphite under high temperature [40], before the formation of out-of-plane disordered sp^3^ bonds. In the following annealing process, the transformation from HDAC to a-D was found. Therefore, amorphous carbons exist in a variety of phase transitions, though the phase diagram is discontinuous.

## CONCLUSION

In summary, six representative 3D amorphous carbons were obtained from MD simulations with EDIP. The uniform simulation method here, independent of a C_60_ precursor, can help us capture comprehensive physical insights into amorphous carbons with a wide density range (0.1–3.4 g/cm^3^). Microstructural topologies are discussed in detail, and computed XRD and S(Q) analysis suggest the simulated amorphous carbons were in line with their experimental counterparts. It is intriguing to present a discontinuous phase diagram in the sp^3^/sp^2^ versus density plane for amorphous carbons, in which the counterintuitive discontinuity originates from their disparate intrinsic microstructural topology. The power law, log(sp^3^/sp^2^) ∝ *ρ*^n^, hints at the intrinsic physics hidden by disordering. Although the phase diagram is discontinuous, multifarious unimaginable phase transitions are found. The discontinuous phase diagram and associated insights hint at many important yet undiscovered intrinsic properties and relationships hidden in non-crystalline carbons.

## Supplementary Material

nwae051_Supplemental_File
