# Supplementary material for: Discontinuous phase diagram of amorphous carbons
Source: Natl Sci Rev. 2024 Feb 6;11(4):nwae051. doi: 10.1093/nsr/nwae051 (PMC10950053; doi:10.1093/nsr/nwae051)
Supplement: nwae051_Supplemental_File [file nwae051_supplemental_file.pdf]

Supplementary data for

Discontinuous phase diagram of amorphous carbons

YinBo Zhu[1,†], ZhouYu Fang[1,†], ZhongTing Zhang[1] and HengAn Wu[1,2,*]

[1]CAS Key Laboratory of Mechanical Behavior and Design of Materials, Department of Modern Mechanics, University of Science and Technology of China, Hefei 230027, China;

[2]State Key Laboratory of Nonlinear Mechanics, Institute of Mechanics, Chinese Academy of Sciences, Beijing 100190, China

†Equally contributed to this work.

***Corresponding author.** E-mail: wuha@ustc.edu.cn

Computational methods and models. All MD simulations were performed using LAMMPS program [1]. The EDIP force field was used in our simulations. The file of the EDIP potential was kindly provided by Prof. Marks [2]. The EDIP potential has proven to be a powerful force field to explore amorphous carbons [3-8]. In the initial model, the simulation box contains 108000 carbon atoms with the FCC lattice, and the initial box length is 129 Å. The periodic boundary conditions were used in all simulations. The timestep is 1 fs. In the beginning, the initial model was relaxed in a 10 ps equilibrium run under 300 K within the NVT ensemble. In this stage, the box size can be gradually changed to obtain models with different densities. Then, the system temperature was increased to a target annealing temperature within 10 ps. Subsequently, annealing simulation was performed under the NPT ensemble, in which the temperature was gradually cooled from the target annealing temperature to 300 K and the pressure was released from the set pressure to zero. Nosé-Hoover thermostat and barostat were used to control the temperature and pressure, respectively. The time of annealing simulations was larger than 2 ns. During the annealing simulations, the annealing temperature and applied pressure were the main variables to obtain different amorphous carbons. During the formation of DGNs, the free carbon atoms after the annealing process should be deleted, and then AIREBO potential [9] was used to obtain final DGNs [8]. It is should be noted that a-DG is hard to be directly obtained from our annealing-MD simulations. We found a-DG phases mainly from the phase transitions of DGNs. More details of simulations of phase transformations are listed in Table S2.

Visual post-processing of amorphous carbons. To identify the microstructural topology of different amorphous carbons, different carbon rings and local ordered microstructures must be discriminated. For sp^2-bonded carbon nanoforms, the polygonal carbon rings (pentagon, hexagon, heptagon, and octagon) were characterized by searching the neighbor list via a "shortest-path" algorithm. The color index of different carbon rings was plotted in Fig. S1. For sp^3-bonded carbon nanoforms, we applied the built-in algorithm in OVITO [10] to identify the number and arrangement of neighboring atoms of each carbon atom, and then the local ordered microstructures can be determined (cubic diamond and hexagonal diamond, and their first- and second-neighbor atoms). The corresponding color index was plotted in Fig. S4.

Calculations of XRD spectra and structural factors (S(Q)). We used the LAMMPS command (compute xrd) to calculate the XRD spectra of different amorphous carbons. The wavelength of the incident ray was 1.541838 Å. The calculation of S(Q) was based on the radial distribution function (g(r)) and Fourier transformation. Since the size of our simulation system is large enough, Fourier transformation of the g(r) can be directly used to obtain the S(Q) curves. The g(r) can be calculated using the LAMMPS command (compute rdf).

Calculation of coordination number (CN). The coordination number of each carbon

atom was counted according to its neighbors with a cutoff of 1.85 Å, and then we can determine the hybrid type of each carbon atom (*sp*, sp^2, and sp^3).

Setups in the simulations of phase transitions. To explore phase transformations among different amorphous carbons, high temperature and high pressure were applied on selected initial amorphous carbons. The setups of simulations in Fig. 4 and Fig. S8 are listed in Table S2.

Table S1. Size parameters in Fig. 1

Name	Density (g/cm^3)	Size of cell length (Å)	Number of atoms
DGN	1.35	116.5	107826
HDAC	2.24	98.8	108000
a-DG	2.69	92.8	108000
a-D	3.17	87.9	108000
p-D	3.24	87.2	108000
NPD	3.36	86.2	108000

Table S2. Simulation setups in Fig. 4 and Fig. S8.

**simulations**	**Fig. 4(a)**	**Fig. 4(b)**	**Fig. S8**
stage I	DGN as the initial structure. The system was relaxed in a 20 ps equilibrium run under 300 K, then the temperature was gradually increased to 4000 K within 10 ps.	DGN as the initial structure. The system was relaxed in a 20 ps equilibrium run under 300 K, then the temperature was gradually increased to 4000 K within 10 ps.	HDAC as the initial structure. The system was relaxed in a 30 ps equilibrium run under 300 K, then the temperature was gradually increased to 4000 K within 10 ps.
stage II	The system was relaxed in a 50 ps equilibrium run under 4000 K, then the pressure was gradually increased to 20 GPa within 10 ps.	The system was relaxed in a 50 ps equilibrium run under 4000 K, then the pressure was gradually increased to 40 GPa within 10 ps.	The system was relaxed in a 200 ps equilibrium run under 4000 K.
stage III	The pressure was fixed at 20 GPa and the temperature was gradually decreased to 300 K within 500 ps.	The pressure was fixed at 40 GPa and the temperature was gradually decreased to 300 K within 500 ps.	The pressure was gradually increased to 40 GPa within 50 ps, then pressure was fixed at 40 GPa

			and temperature was gradually decreased to 300 K within 500 ps.
stage IV	The pressure was gradually decreased to 0 GPa within 10 ps, then the system was relaxed in a 50 ps equilibrium run.	The pressure was gradually decreased to 0 GPa within 10 ps, then the system was relaxed in a 50 ps equilibrium run.	The pressure was gradually decreased to 0 GPa within 50 ps, then the system was relaxed within a 50 ps equilibrium run.

Table S3. Experimental data* in the phase diagram (Fig. 3)

References	Density (g/cm^3)	Fraction of sp^2 bonds	Fraction of sp^3 bonds
[11]	3.11	5.2%	--
[12]	3.25	--	80.5% (±8%)
	3.3	--	95.1% (±1.7%)
[13]	2	--	9% (±2%)
	2.5	--	22% (±5%)
[14]	1.4	94.4% (3.2%)	--
[15]	1.6	95%	--
	1.88	96.73%	--
	1.77	98.66%	--
	1.55	99.58%	--
	1.52	99.91%	--
	1.51	99.95%	--
[16]	2.39	--	22.686%
	2.48	--	34.535%
	2.28	--	36.94%
	2.87	--	68.84%
	2.943	--	71.756%
	2.937	--	75.59%
	2.96	--	76.42%
	2.867	--	75.91%
	2.83	--	77.98%
	3.03	--	80.897%
	3.12	--	84.846%
	3.135	--	86.915%
	3.17	--	85.88%
	3.224	--	87.855%
[17]	2.6	--	~20%
	2.8	--	~50%

3.1	--	~70%

*In some references, most data of density and fraction of sp^2 (or sp^3) bonds in experiments are estimated. In Refs. [15-16], the data are extracted from figures.

Reference

[1] N. A. Marks, Physical Review B **63**, 035401 (2000).

[2] C. de Tomas, A. Aghajamali, J. L. Jones, D. J. Lim, M. J. López, I. Suarez-Martinez, and N. A. Marks, Carbon **155**, 624 (2019).

[3] C. de Tomas, I. Suarez-Martinez, and N. A. Marks, Carbon **109**, 681 (2016).

[4] C. de Tomas, I. Suarez-Martinez, F. Vallejos-Burgos, M. J. López, K. Kaneko, and N. A. Marks, Carbon **119**, 1 (2017).

[5] C. d. Tomas, I. Suarez-Martinez, and N. A. Marks, Applied Physics Letters **112**, 251907 (2018).

[6] J. W. Martin, C. de Tomas, I. Suarez-Martinez, M. Kraft, and N. A. Marks, Physical Review Letters **123**, 116105 (2019).

[7] Y. Zhu, Y. Wang, B. Wu, Z. He, J. Xia, and H. Wu, Nano Letters **21**, 8401 (2021).

[8] Y. Wang, Y. Zhu, and H. Wu, Physical Chemistry Chemical Physics **23**, 10290 (2021).

[9] S. J. Stuart, A. B. Tutein, and J. A. Harrison, Journal Chemical Physics 2000, **112**, 6472 (2000).

[10] A. Stukowski, Modelling and Simulation in Materials Science and Engineering **18**, 015012 (2010).

[11] H. Tang *et al.*, Nature **599**, 605 (2021).

[12] Z. Zeng *et al.*, Nature Communications **8**, 322 (2017).

[13] M. Hu *et al.*, Science Advances **3**, e1603213 (2017).

[14] X. Zhang, L. Zhong, A. Mateos, A. Kudo, A. Vyatskikh, H. Gao, J. R. Greer, and X. Li, Nature Nanotechnology **14**, 762 (2019).

[15] K. Jurkiewicz, M. Pawlyta, D. Zygadło, D. Chrobak, S. Duber, R. Wrzalik, A. Ratuszna, and A. Burian, Journal of Materials Science **53**, 3509-3523 (2018).

[16] A. LiBassi, A.C. Ferrari, V. Stolojan, B.K. Tanner, J. Robertson, and L.M. Brown, Diamond and Related Materials **9**, 771-776 (2000).

[17] Z. Li *et al.*, Nature Materials, **22**, 42-49 (2023).

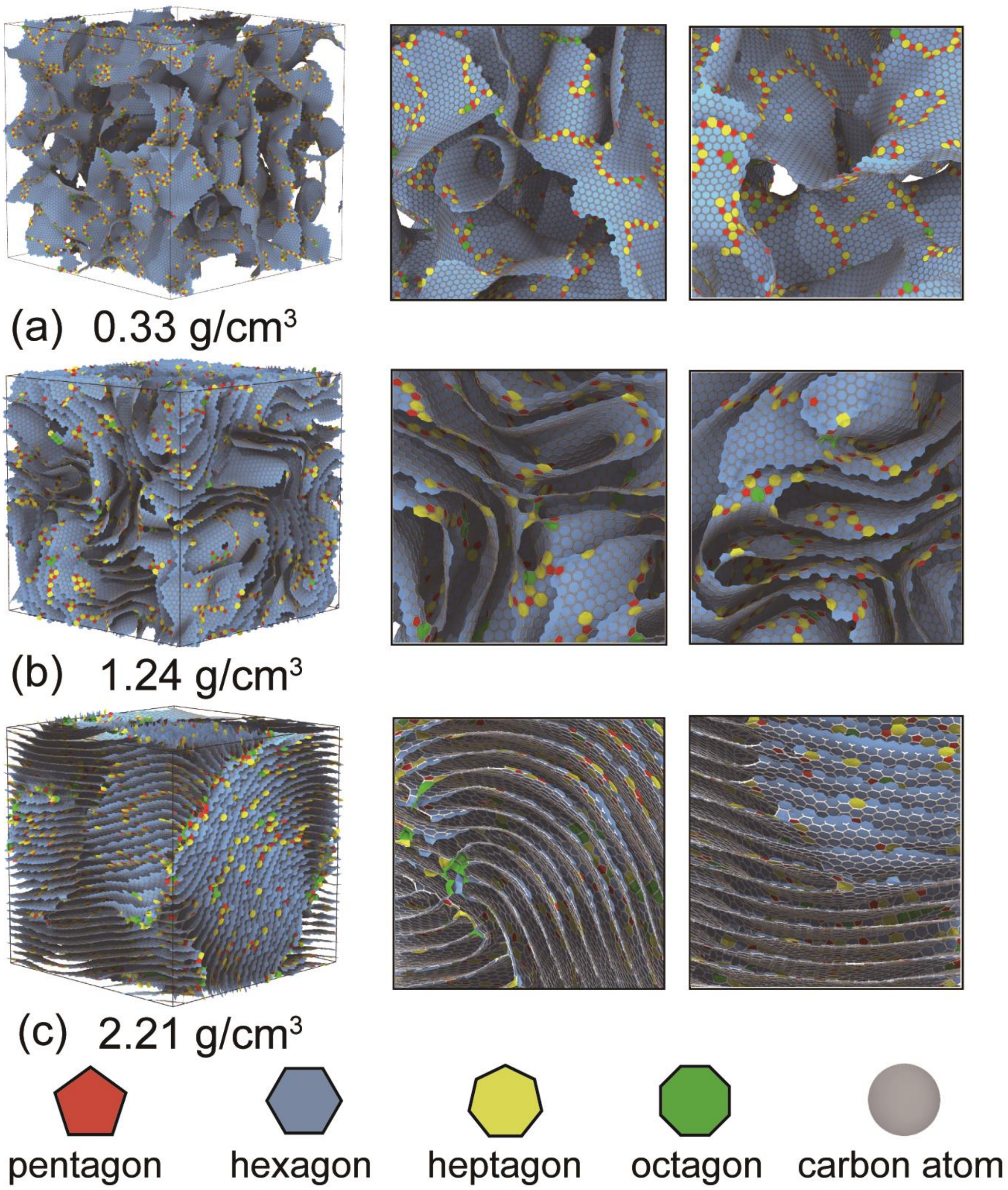

Figure S1. Microstructural topology of DGNs with different densities. (a) Low-density porous carbons ($\rho < 0.6$ g/cm^3). (b) Medium-density disordered carbons (0.6 g/cm^3 $< \rho <$ 1.8 g/cm^3). (c) High-density amorphous graphite-like carbons (1.8 g/cm^3 $< \rho <$ 2.4 g/cm^3). The framework of DGNs can well depict the topology of the three different carbons, though their microstructures and density are vastly different.

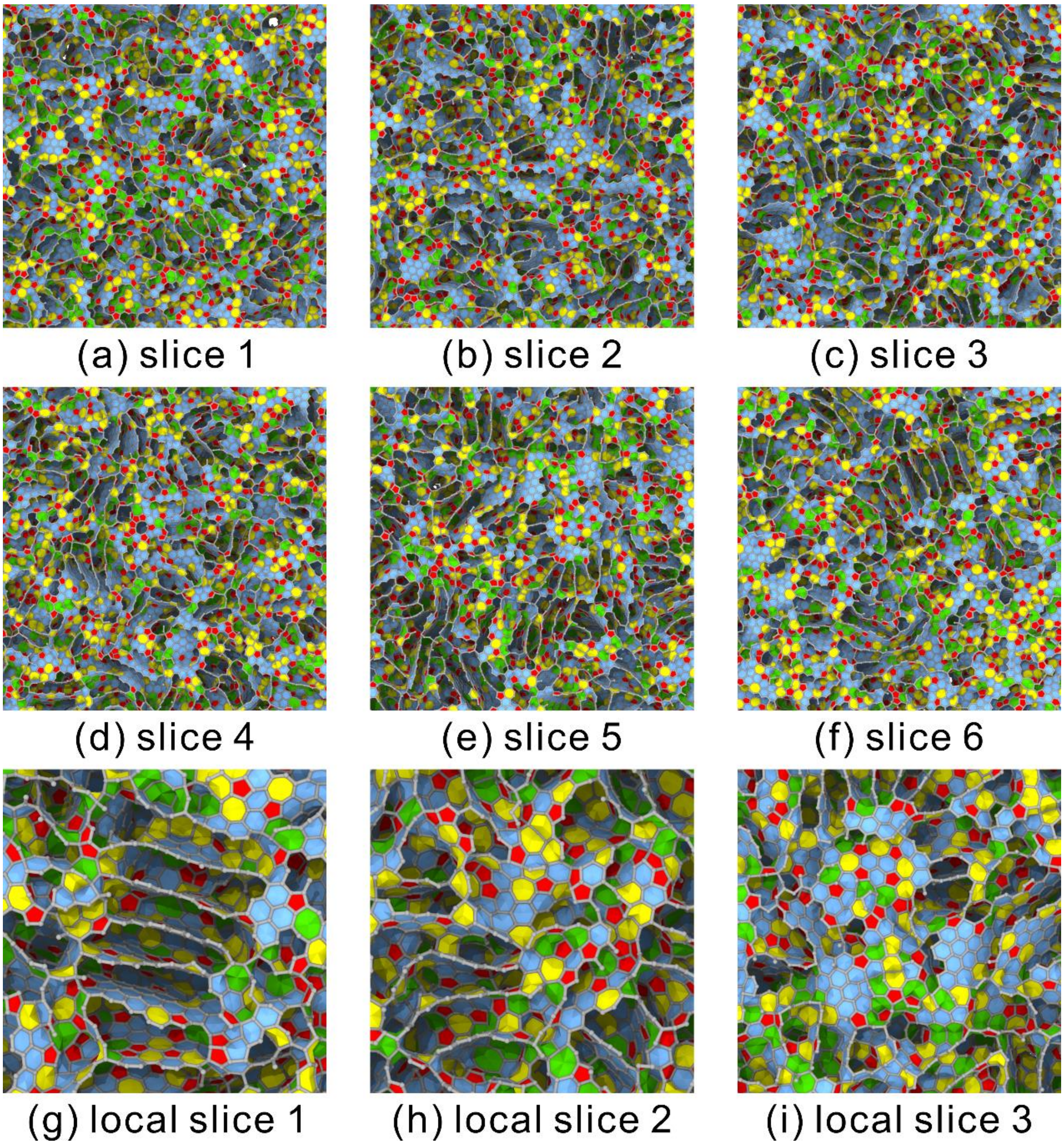

Figure S2. Microstructural topology of HDACs. HDAC is a more disordered sp^2-dominated amorphous carbon. HDACs exhibit more out-of-plane topological defects compared to DGNs, resulting in more non-hexagonal carbon rings and small-size fragments. One important feature here is the nanometer-sized amorphous graphite clusters, highlighted in (g-i), providing the short-range order in HDACs. Polygonal carbon rings in HDACs are more distorted, and the characteristic size of fragments is only several carbon rings. Although the microstructure of HDAC is highly disordered, the fragments maintain well 3D connection through hybrid sp^2-sp^3 nanoforms. Considering the high density and a variety of nanopores in microstructures, HDACs can be regarded as a close-cell material with atomically disordered nanoscale cell walls.

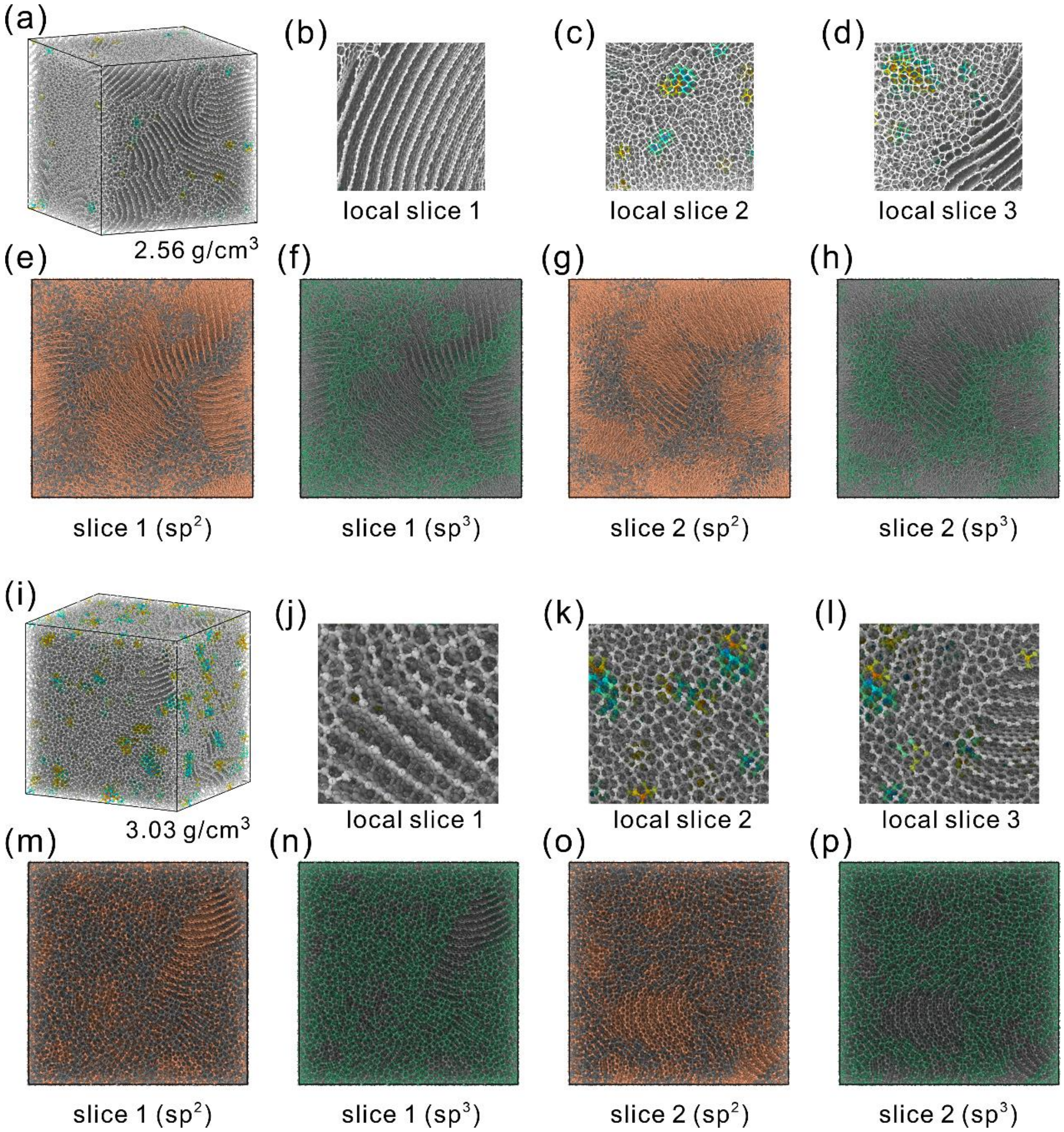

Figure S3. Microstructural topology of amorphous diaphite (a-DG). (a) a-DG (ρ = 2.56 g/cm^3). (b) Local graphite structure. (c) Local sp^3-bonded structure with diamond-like nuclei. (d) Local hybrid sp^2-sp^3 structure. (e) Slice 1 highlighting the sp^2 bonds (color in yellow). (f) Slice 1 highlighting the sp^3 bonds (color in cyan). (g) Slice 2 highlighting the sp^2 bonds (color in yellow). (h) Slice 2 highlighting the sp^3 bonds (color in cyan). (i) a-DG (ρ = 3.03 g/cm^3). (j) Local graphite structure. (k) Local sp^3-bonded structure with diamond-like nuclei. (l) Local hybrid sp^2-sp^3 structure. (m) Slice 1 highlighting the sp^2 bonds (color in yellow). (n) Slice 1 highlighting the sp^3 bonds (color in cyan). (o) Slice 2 highlighting the sp^2 bonds (color in yellow). (p) Slice 2 highlighting the sp^3 bonds (color in cyan). With the increase of density, a few diamond-like nanonuclei can be found in a-DGs. While sp^3-bonded atoms in a-DGs only exhibit short-range order, the medium-range order is provided by amorphous graphite nanograins.

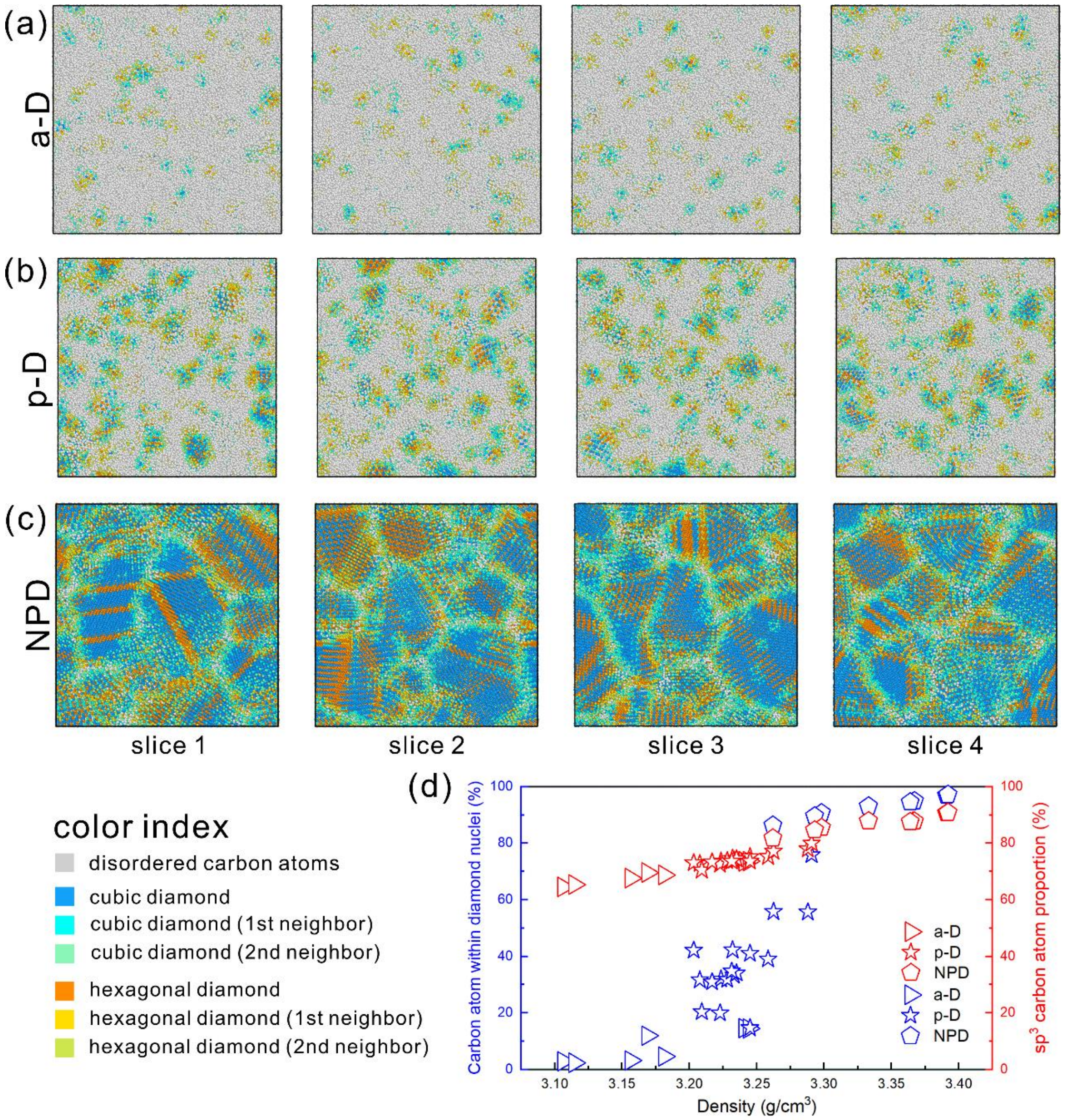

Figure S4. Microstructural topology of ultrahigh-density amorphous carbons. (a) Diamond-like nanonuclei in a-D. (b) Diamond-like nanonuclei in p-D. (c) Diamond nanograins in NPD. (d) The fraction of carbon atoms within diamond nuclei and fraction of sp3-bonded carbon atoms as functions of density. Here, the diamond-like nanobuclei and nanograins include the first- and second-neighbor of diamond, in which a few sp^2-bonded carbon atoms (CN=3) are found. Thus, for NPDs, the fraction of carbon atoms in diamond nuclei are slightly larger than that of sp^3-bonded carbon atoms.

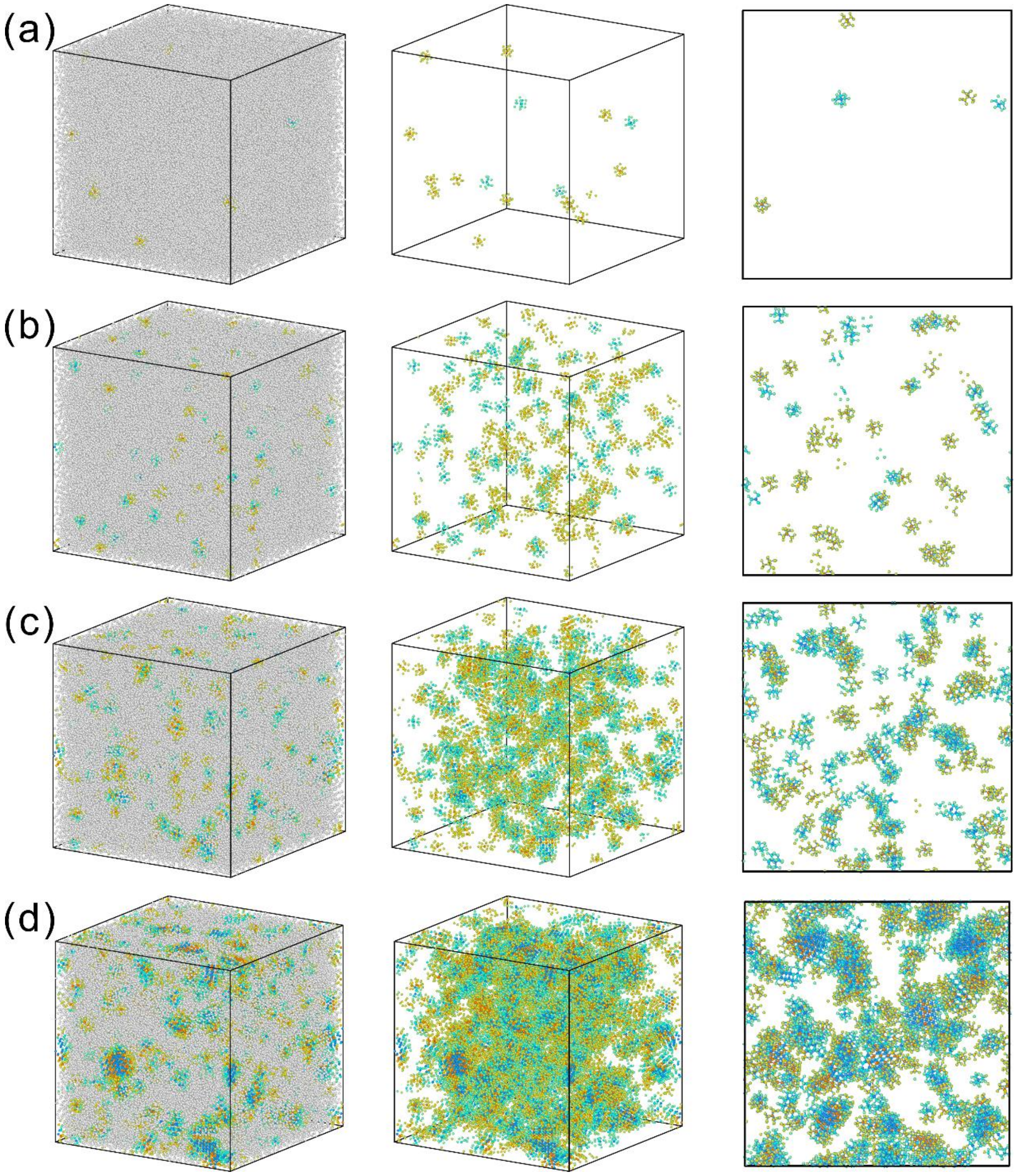

Figure S5. Nucleation in p-D. (a) 20 ps. In the beginning of cooling process, random diamond-like nanonuclei appeared in high-temperature liquid carbon atoms. But those unstable nanonuclei are annihilated in the melting liquid carbon at any time. (b) 130 ps. More random diamond-like nanonuclei appeared in the liquid carbon atoms. Those nanonuclei are still unstable, though the characteristic size is slightly large. (c) 780 ps. Stable diamond-like nanonuclei arose in the system. Then, nucleating growth and epitaxial growth of nanonuclei were found in the system. (d) Final stale p-D. After a long-time cooling process, stable p-D with high volume fraction of paracrystallites was obtained. Gray atoms denote disordered carbon atoms. The thickness of slab snapshots in the third column is 20 Å. The temperature-dependent paracrystalline nucleation in atomically disordered diamonds was discussed in our recent study (Nano Lett. 2024, 24, 312-318).

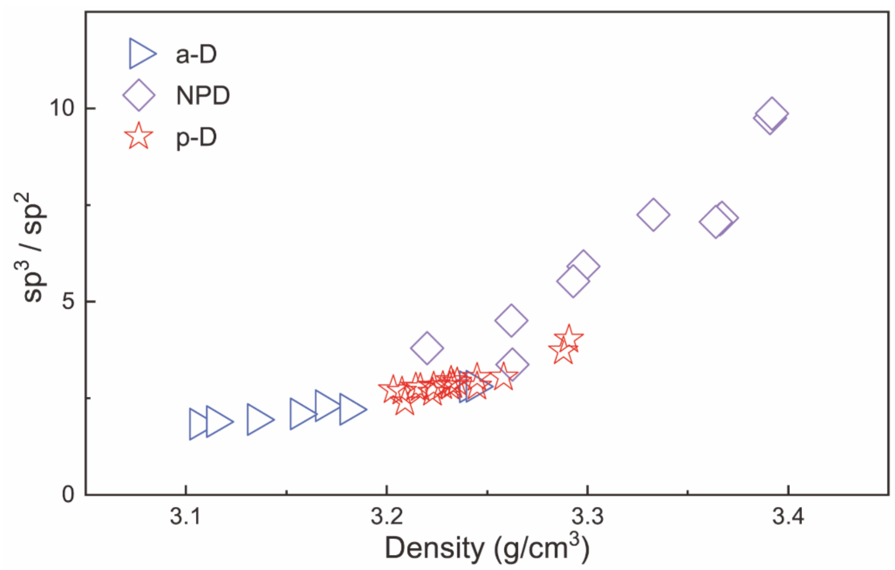

Figure S6. Detailed view of density distributions of a-D, p-D, and NPD in Fig. 3. For p-D with high volume percentage of paracrystallites, coordinated high-pressure and high-temperature conditions are needed to promote more sp^3-bonded atoms that transform into the first- and second-neighbor of diamond-like nuclei. Otherwise, the continuous nucleation process would result in the transformation from p-D to NPD.

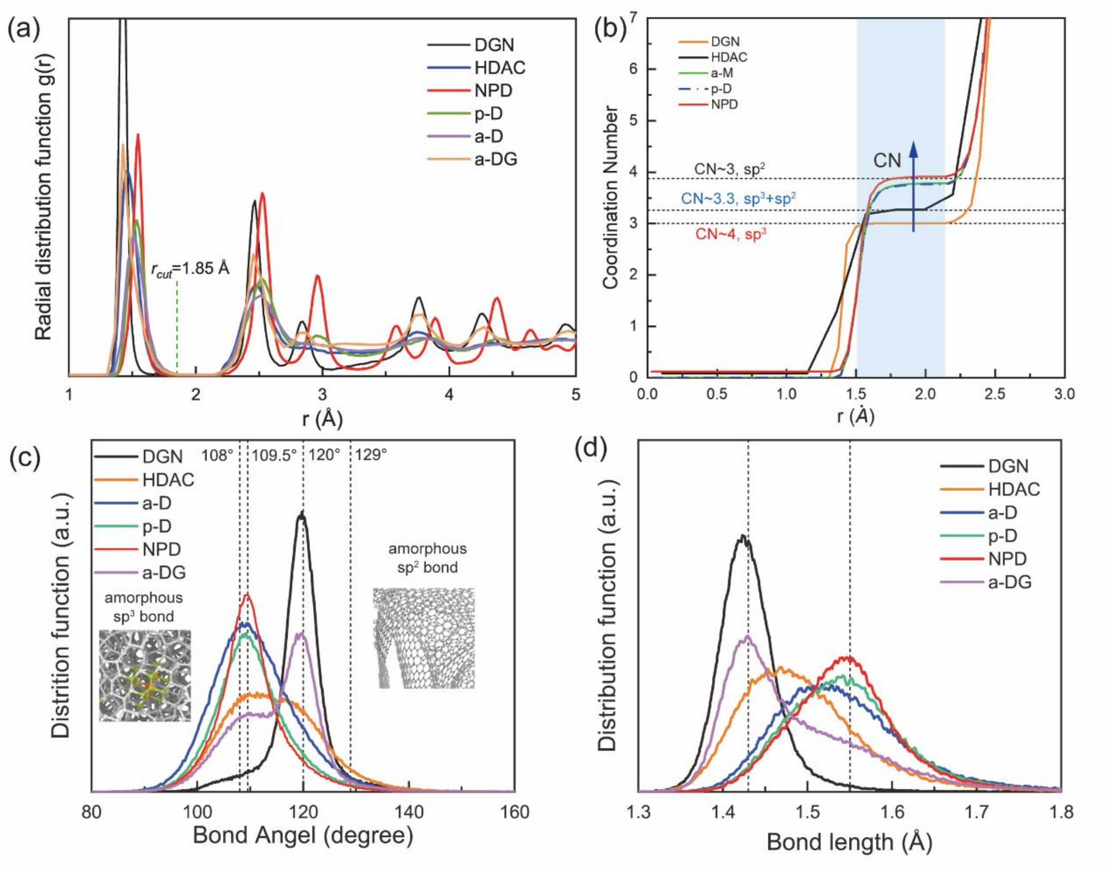

Figure S7. Microstructural characterizations of different amorphous carbons obtained in MD simulations. (a) Radial distribution function. (b) Coordinate number. (c) Distribution of bond angle. (d) Distribution of bond length.

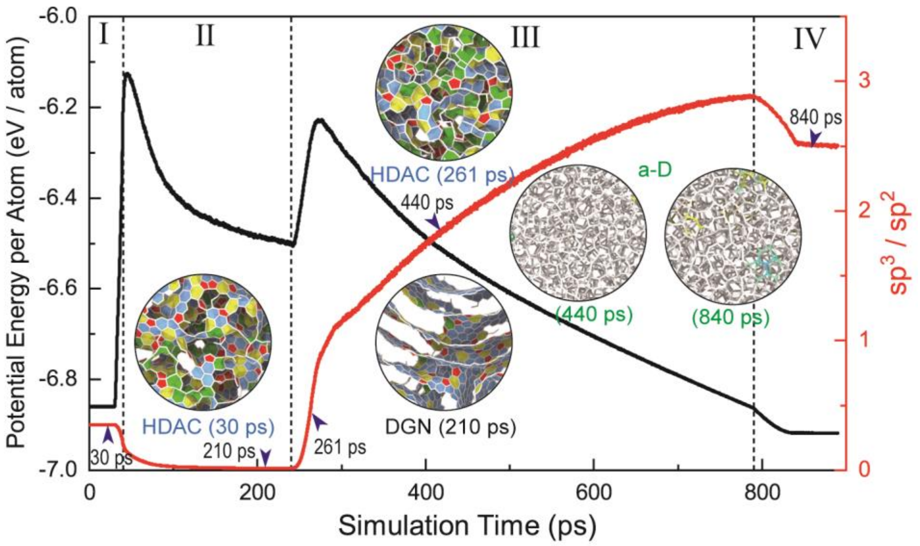

Figure S8. Reversible phase transitions between the HDAC and high-density DGN (amorphous graphite), and phase transformation from HDAC to a-D. The dark and reds lines denote the potential energy (per atom) and sp^3/sp^2 value as functions of the simulation times. The insets highlight the microstructures at different stages. In the stage I (0-40 ps), the initial HDAC phase was relaxed in a 30 ps equilibrium run under 300 K, and then the temperature increased to 4000 K within 10 ps. In the stage II (40-240 ps), the system was relaxed under 4000 K within 200 ps. In the stage III (240-780 ps), the pressure increased to 40 GPa within 50 ps, and then the system temperature decreased from 4000 K to 300 K within 500 ps. In the stage IV (780-880 ps), the pressure decreased from 40 GPa to 0 GPa within 50 ps, and then the system was relaxed in a 50 ps equilibrium run. It should be noted that the potential energy in the stage II shows an obvious drop, indicating that the phase transition from HDAC to high-density DGN (amorphous graphite) under 4000 K is a spontaneous process.
